# Selective recognition of mitochondrial acyl carrier protein by the regulatory protein LYRM2

**DOI:** 10.1016/j.jbc.2026.113422

**Published:** 2026-08-10

**Authors:** Yixing Suo, James J. La Clair, Michael D. Burkart

**Affiliations:** Department of Chemistry and Biochemistry, University of California, San Diego, California, USA

**Keywords:** mitochondrial acyl carrier protein, mitochondrial fatty acid synthesis, LYRM protein, acyl-chain selectivity, mitochondrial metabolism

## Abstract

Human mitochondrial fatty acid synthesis plays a central role in coordinating lipid metabolism with respiratory chain biogenesis through the central mitochondrial acyl carrier protein (mACP). Although several Leucine-Tyrosine-Arginine motif (LYRM) family mitochondrial regulatory proteins have been shown to associate with mACP, the structure or function of most of them has remained undefined. Here, we identify one of the regulatory proteins, LYRM2, as an acyl-chain selective mACP-binding protein. Systematic expression screening of underexplored regulatory proteins revealed LYRM2 as uniquely stable in isolation. Gel electrophoresis analysis demonstrated specific complex formation between LYRM2 and acylated mACP. Biophysical and analytical analyses showed preferential binding to long-chain acyl-mACP species, with strongest interactions observed for C12. These results establish LYRM2 as a selective mACP-interacting protein and support a role for acyl-state recognition in mitochondrial regulatory signaling.

Mitochondrial fatty acid synthesis (mtFAS) has emerged as a regulatory platform that links mitochondrial lipid metabolism to respiratory function and overall oxidative capacity (1, 2, 3). Central to this pathway is mitochondrial acyl carrier protein (in humans, mACP), which not only shuttles intermediates but also serves as a protein-interaction scaffold that couples mtFAS to oxidative phosphorylation (OXPHOS) and iron-sulfur (Fe-S) cluster biogenesis (4, 5, 6, 7). Fe-S clusters are required for the assembly and activity of electron transport chain complexes I, II, and III, which are essential for energy production (8, 9). Through its acyl-dependent protein interactions, mACP occupies a critical position for transmitting metabolic state information into mitochondrial regulatory pathways (10).

A growing group of small mitochondrial regulatory proteins, including several Leu-Tyr-Arg motif (LYRM) family members, has been identified as mACP-associated factors (Fig. 1) (11, 12). LYRM proteins are typically small, mainly α-helical, non-enzymatic adaptors defined by a conserved N-terminal Leu-Tyr-Arg sequence. Structural and biochemical studies of LYRM3/NDUB9, LYRM4/ISD11, LYRM6/NDUA6, and L0R8F8 have established their roles in respiratory complex biogenesis and Fe–S cluster metabolism, supporting a model in which LYRM proteins act as molecular adaptors for mACP-mediated regulation (13, 14, 15, 16, 17, 18). However, a substantial fraction of predicted mACP-associated regulatory proteins remains structurally and functionally uncharacterized (Table S1). Among these, LYRM2 has been identified as an mACP-associated protein and linked to complex I assembly in large-scale mitochondrial protein interaction mapping and subsequent proteomic studies, yet its direct biochemical activity and the mechanistic basis of its interaction with mACP remain undefined (10, 19, 20).

**Figure 1:**
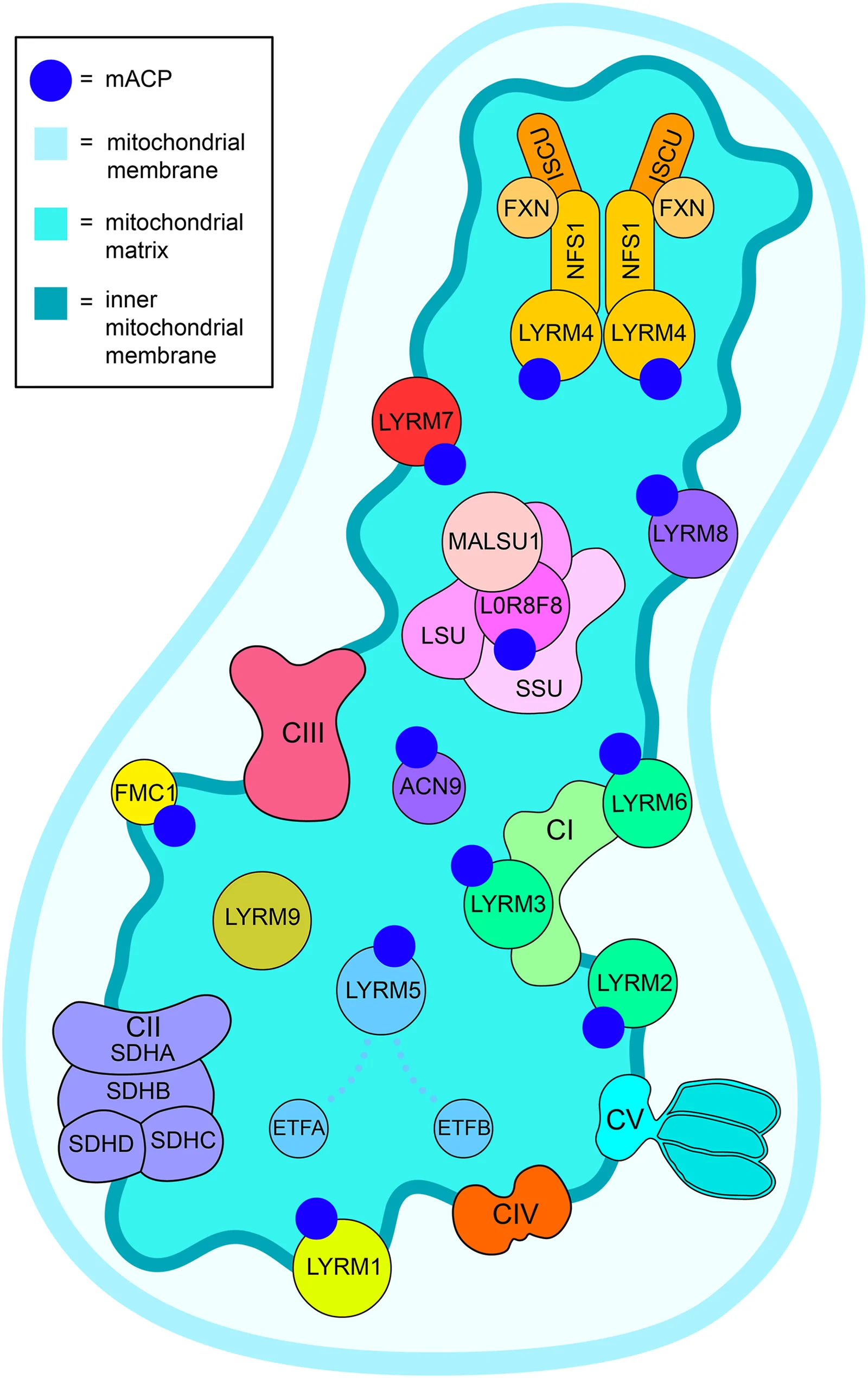
**Illustration of the multiple binding partners engaged by mACP within thehumanmitochondria**. Protein complexes are observed on the inner mitochondrial membrane or in the mitochondrial matrix. Further information is provided in Table S1.

Here, we performed a systematic expression and interaction analysis of underexplored mACP-associated regulatory proteins and identified LYRM2 as a specific acyl-mACP binding partner. LYRM2 is uniquely amenable to independent recombinant expression and forms a defined complex with acylated mACP in an acyl-chain dependent manner. These findings provide the first direct biochemical characterization of the LYRM2-mACP interaction and support a role for acyl-state recognition in mtFAS-dependent mitochondrial regulation.

## Results and discussion

### LYRM2 expression and purification

To identify mitochondrial regulatory proteins that may participate in mACP-dependent signaling and regulation, we cloned a focused panel of candidates predicted or reported to associate with mACP (Table S2). Coding sequences corresponding to LYRM1 (Uniprot Q43325, Gene *LYRM1*), LYRM2 (Uniprot Q9NU23, Gene *LYRM2*), LYRM5 (Uniprot Q6IPR1, Gene *ETFRF1*), LYRM7 (Uniprot Q5U5X0, Gene *LYRM7*), LYRM8 (Uniprot A6NFY7, Gene *SDHAF1*), ACN9 (Uniprot Q9NRP4, Gene *SDHAF3*), and FMC1 (Uniprot Q96HJ9, Gene *FMC1*) were synthesized without their mitochondrial targeting peptides and inserted into pET-28 vectors encoding N-terminal His_6_ tags.

Initial recombinant expression trials in *Escherichia coli* revealed a slight divergence in solubility among the proteins (Fig. S1). Of the seven proteins examined, LYRM2 and ACN9 were uniquely capable of independent expression, despite relatively low overall expression levels, with most forming insoluble inclusion body. In contrast, the remaining constructs exhibited poor to no expression, consistent with the emerging view that many mitochondrial regulatory proteins are obligatory partners that depend on complex formation with mACP for stability. In support of this model, co-expression experiments using co-transformation with an mACP construct substantially improved the apparent expression and solubility of several candidates, suggesting that mACP functions as a stabilizing partner for multiple regulatory proteins (Fig. S2). Given its unique productivity and the absence of any prior biochemical characterization, LYRM2 was selected for further analysis. We next optimized LYRM2 expression across multiple *E. coli* strains and induction conditions. Although overall yields remained modest, expression in *E. coli* BL21(DE3) under induction at 16 °C for 16 to 18 h produced the highest levels of soluble protein and was therefore adopted for subsequent studies. nickel-nitrilotriacetic acid (Ni-NTA) affinity chromatography, followed by size-exclusion chromatography, provided the purified LYRM2, which was verified using mass spectrometry (Fig. S3).

### Selectivity of LYRM2 complexation with ACPs

**Figure 2:**
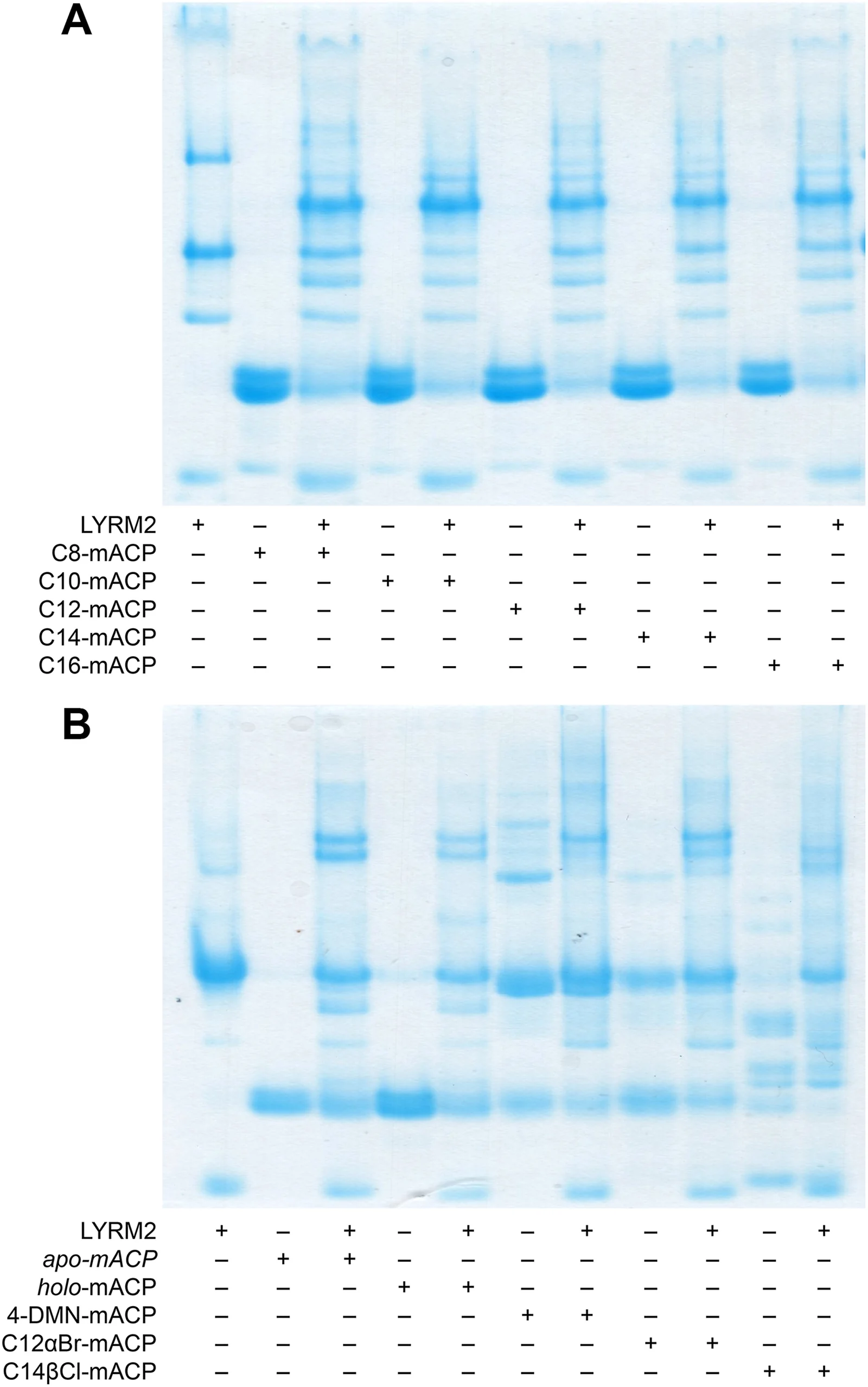
**Native-PAGE analysis of acyl-dependent complex formation between LYRM2 and mACP**. *A*, 12% native-PAGE of LYRM2 incubated for 1 h at 23 °C with a panel of acyl-mACP species (C8, C10, C12, C14, and C16) shows the appearance of a new band consistent with formation of discrete LYRM2·acyl-mACP complexes. *B*, Under identical conditions, no comparable shifted band is observed when LYRM2 is incubated with apo-mACP, holo-mACP, or non-native substrate/probe-tethered mACP species.

To demonstrate the interaction between LYRM2 and mACP, we examined complex formation using a series of gel electrophoresis assays. Incubation of LYRM2 with acylated mACP species bearing C8-C16 acyl chains at 23 °C for 1 h resulted in the appearance of new bands indicating the formation of discrete LYRM2·mACP complexes. These shifted species were observed reproducibly across native and urea polyacrylamide gel systems (Fig. 2*A*, Fig. S4), while SDS-PAGE analysis confirmed the expected molecular weights of the individual LYRM2 and mACP components (Fig. S5). The multiple native bands observed for LYRM2 likely reflect distinct oligomeric or conformational states under non-denaturing conditions, as all species reproducibly formed the corresponding shifted complex upon incubation with acyl-mACP. Together, these data indicate that the LYRM2·mACP interaction is sufficiently stable to persist under both native and mildly denaturing conditions. Notably, no comparable band formation was detected when LYRM2 was incubated with *apo-*, *holo-*, or any non-native substrate-tethered mACPs, including fluorophore-based or substrate-mimic probes (Fig. 2*B*, Fig. S6). This result demonstrates that complex formation is not a promiscuous consequence of phosphopantetheine flipping but instead depends on the specific acyl-mACP context. These data establish LYRM2 as a mACP-interacting protein and suggest that the acyl substrate contributes to molecular recognition.

**Figure 3:**
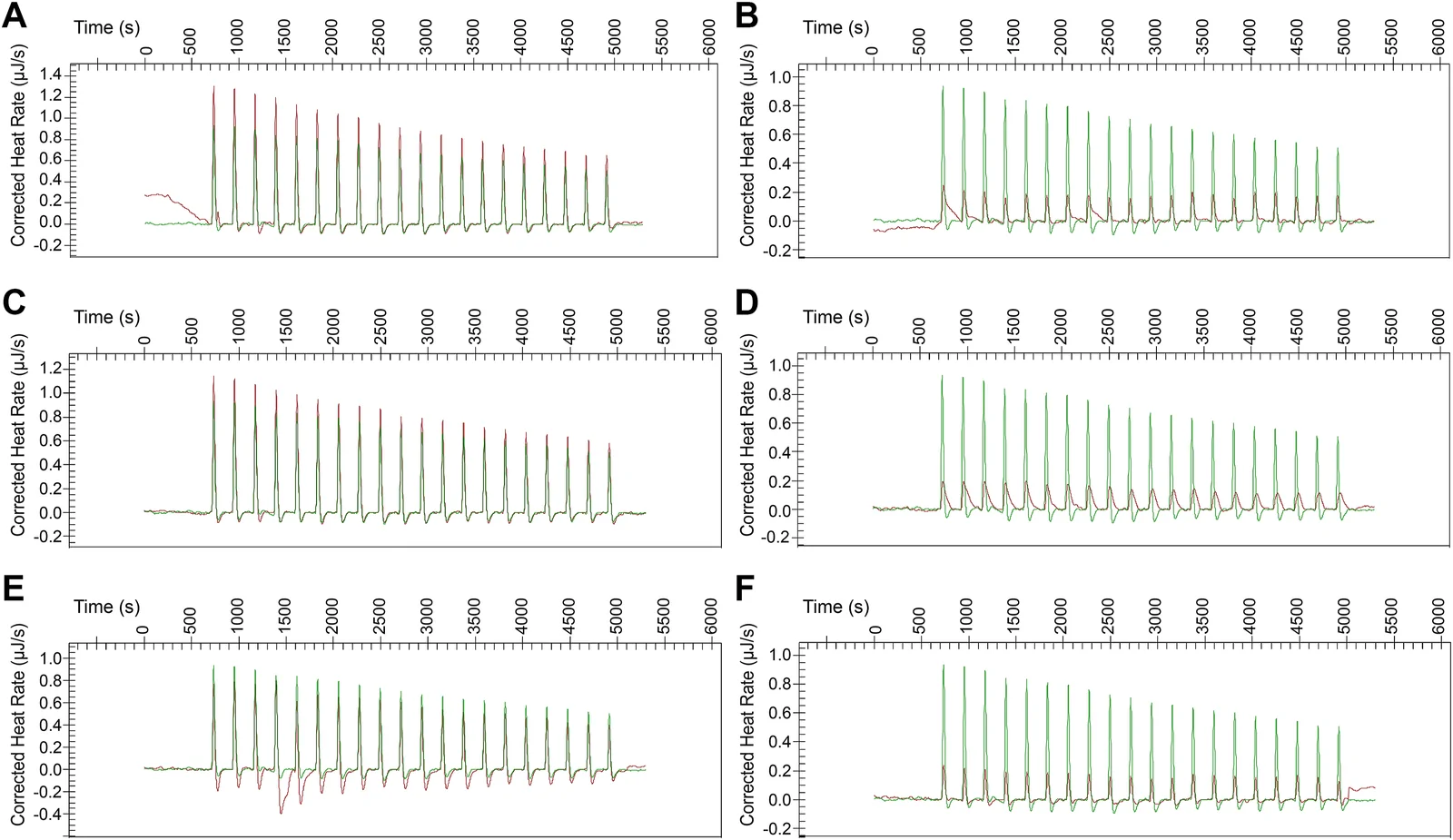
**Representative ITC injection traces for LYRM2 titrated into the acyl-mACP series**. *A*, C8-mACP. *B*, C10-mACP. *C*, C12-mACP. *D*, C14-mACP. *E*, C16-mACP. *F*, malonyl-mACP. LYRM2-into-acyl-mACP runs (*red*) are overlaid against the LYRM2-into-buffer blank (*green*).

We next sought to quantify LYRM2’s selectivity across the acyl-mACP series. Isothermal titration calorimetry (ITC) experiments were performed in duplicate by titrating 0.1 mM LYRM2 into 0.01 mM acyl-mACPs, with LYRM2-into-buffer injection used as a blank control. As LYRM2·mACP binding is expected to be 1:1, we fit the data primarily with the stoichiometry fixed (n = 1) to enable direct comparison of apparent affinities across substrates (Fig. 3, Fig. S7), while fits with n free were used as a diagnostic of data quality. In the injection plots, the blank trace (green) provides the heat-of-dilution/baseline reference, and the binding experiment (red) is expected to show a deviation from this background. However, for several substrates, the red trace is weaker than the corresponding blank signal, indicating that the apparent heats are dominated by dilution/mixing effects and baseline subtraction uncertainty rather than robust binding enthalpy. Consistent with this, the two runs provided the basis for quantitative comparisons, with C12-mACP giving the most consistent dataset with apparent K_d_ values of 2.64 × 10^−6^ and 2.37 × 10^−6^ M (Table S3). By contrast, other substrates remained more variable between runs or showed poor signal-to-blank separation. All acyl-mACP species, including long-chain mACP, remained soluble throughout purification and ITC measurements, and no evidence of precipitation or aggregation was observed. We therefore attribute the weaker calorimetric signals primarily to their limited separation from the background heat rather than differences in sample quality. Notably, although C14-mACP returned the smallest apparent K_d_ and a very small run-to-run standard deviation, the underlying injection traces show limited separation from the blank, indicating that this dataset is not reliably interpretable as the tightest interaction in the series. Taken together, these analyses support acyl-chain dependent recognition by LYRM2 while highlighting C12 as the most reproducible dataset in this panel and the most reliable benchmark for comparing apparent affinities under our conditions.

Beyond its canonical biosynthetic role, ACP is also known to participate in nutrient-responsive regulatory processes in bacterial systems. In *E. coli*, the stringent response enzyme bifunctional (p)ppGpp synthase/hydrolase, SpoT, has been shown to physically associate with the *E. coli* ACP (AcpP), linking fatty acid metabolism to SpoT regulatory activity (21, 22). Importantly, SpoT has not been demonstrated to exhibit strict specificity for a single defined acyl species but is instead sensitive to the ratio of unacylated *versus* acylated AcpP to respond to changes in the cellular AcpP pool (23). In contrast, LYRM2 does not show tight interaction with malonyl-mACP and preferentially associates with long-chain acyl-mACP species, indicating that LYRM2 engages ACP through a distinct, more acyl-state selective biochemical interaction.

To further assess whether the experimentally observed acyl-chain preference was consistent with structural modeling, we modeled LYRM2 in complex with acyl-mACP using the Boltz-2 model that extends AlphaFold3 architecture to predict 3D complex structures and binding affinities. Scanning the even-carbon acyl series (C8-C16), the predicted interaction is confidently modeled at every chain length and shows a reproducible, non-monotonic dependence on acyl chain length. Although these models do not establish the molecular basis of substrate recognition, they independently predicted a preference for long-chain acyl species, with C12 exhibiting the most favorable predicted affinity, corroborating our direct experimental studies (Figs. S7-S8, Table S4-S5).

Whether LYRM2 is capable of directly recognizing free fatty acids in the absence of mACP also remains an interesting question. Our data suggest that the native mACP scaffold is required for productive complex formation, although further studies will be needed to determine whether LYRM2 possesses intrinsic fatty acid-binding activity independent of mACP.

### *In vivo* acyl chain selectivity of LYRM2

**Figure 4:**
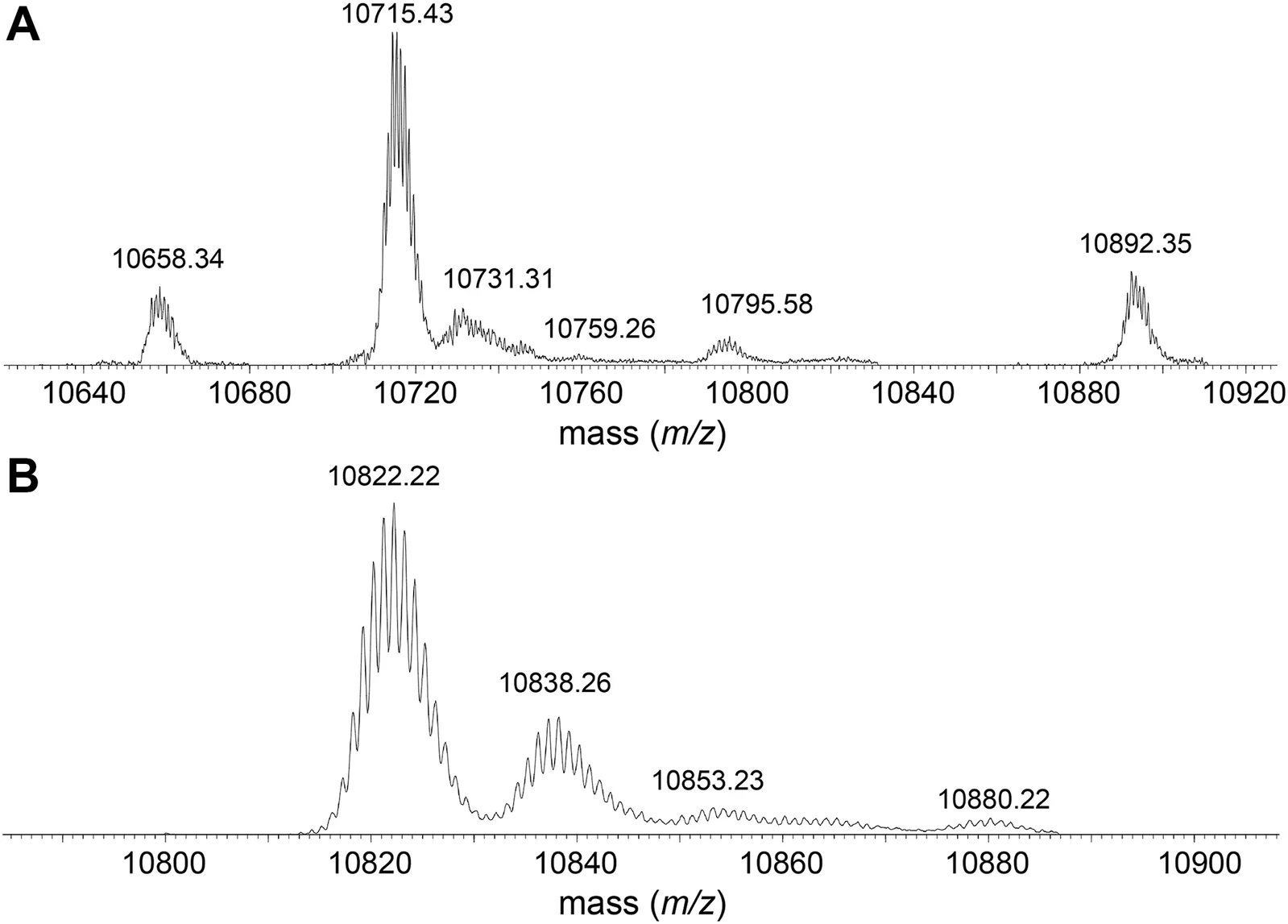
**ESI-TOF mass spectra of His6-LYRM2/mACP co-expression**. *A*, His_6_-LYRM2 appears at 10,715.43, with a +178 Da adduct at 10,892.35 consistent with α-N-6-phosphogluconoylation of the His-tag in *E. coli*. *B*, Deconvoluted peaks consistent with acyl-mACP are observed at 10,795.58 (in A) and 10,822.22 (assigned to C10-C12), with minor higher-mass species at 10,853.23 and 10,880.22 (assigned to C14-C16). Peaks at 10,658.34 and 10,838.26 are consistent with additional N-terminal Gly removal of LYRM2 and its corresponding +178 Da adduct.

To evaluate whether the acyl-chain selectivity observed *in vitro* is reflected under more physiologically relevant conditions, mACP was co-expressed in *E. coli* in the presence of LYRM2 and analyzed by electrospray ionization time-of-flight mass spectrometry (ESI-TOF MS). This approach enables direct assessment of the acyl-mACP population when interacting with LYRM2 during expression and post-translational modification. Under these conditions the spectra were dominated by LYRM2, appearing primarily as the N-terminal methionine excised species (24), with a portion of α-N-6-phosphogluconoylation of His-tag in *E. coli* (25), but additional ions attributable to mACP were also detected in the co-eluting fractions. The mACP-associated signals were enriched for long-chain acylated forms, with masses most consistent with C10-C12 modification and only minor contributions from C14-C16 species (Fig. 4). Notably, prolonged incubation of the LYRM2·mACP complex in the presence of SDS did not result in detectable dissociation, and the acyl substrate remained intact throughout the experiment, with the unchanged product profile indicating that the interaction is unusually stable once formed. This stability contrasts with the behavior of the LYRM4 (ISD11)·mACP complex, which readily denatures and unfolds upon SDS treatment. The persistence of the LYRM2·mACP complex suggests that LYRM2 engages acyl-mACP through a particularly robust interaction, consistent with its proposed role as a selective regulatory binding partner rather than a transient assembly factor. Because the endogenous acyl-mACP distribution in the *E. coli* expression system may contribute to the observed acyl profile, these experiments do not distinguish whether LYRM2 actively influences fatty acid biosynthetic output or preferentially associates with pre-existing acyl-mACP species. Nevertheless, the enrichment of C12-mACP is consistent with the acyl-chain preference observed in the native PAGE and ITC experiments and therefore provides independent supportive evidence for acyl-state dependent recognition by LYRM2.

## Conclusion

Collectively, these results identify LYRM2 as a previously unrecognized, acyl-chain selective mACP-binding protein. The preference for C12 acyl species is consistent with the previously published ISD11-mACP structure, suggesting that LYRM2 is positioned to sense or stabilize discrete intermediates, broadening the mechanisms by which mACP communicates metabolic state information to mitochondrial regulatory pathways. This work establishes a biochemical foundation for LYRM2 and supports a more comprehensive model in which LYRM proteins act as acyl-state responsive adaptors within the mitochondrial signaling network. Although LYRM2 has been implicated in complex I assembly through proteomic and genetic studies, few direct interaction partners beyond mACP have been experimentally established. The biochemical characterization presented here therefore provides a foundation for future studies aimed at defining how LYRM2 cooperates with the complex I assembly machinery. Because the present study is limited to the biochemical characterization of the LYRM2-mACP interaction, additional cellular studies will be required to determine how this interaction contributes to mitochondrial regulation under physiological conditions.

Interestingly, although the core mtFAS enzymes are evolutionarily related to their bacterial counterparts, current evidence suggests that LYRM2 is largely restricted to eukaryotes, and no orthologs have been reported in bacteria, including α-proteobacteria. This evolutionary distribution suggests that LYRM2 may represent a eukaryotic adaptation that links the ancestral mtFAS machinery to mitochondrial regulatory pathways (26).

In summary, the preferential association of LYRM2 with acyl-mACP centers on C12 intermediates, indicating that LYRM2 is unlikely to function as a constitutive structural component and instead supports a role in acyl-state dependent regulation. These findings expand the functions of LYRM-family mitochondrial regulatory proteins, which are increasingly seen as dynamic adaptors that link mtFAS-derived signals to diverse mitochondrial pathways. Together, these biochemical observations further support a model in which acyl-chain identity serves as a molecular cue that modulates mitochondrial processes through protein-protein interactions. This provides a biochemical foundation for future investigations into how mtFAS-derived metabolic states are translated into mitochondrial regulatory responses.

## Experimental procedures

For general materials and methods, protein expression and purification procedures, and Boltz-2 covalent docking, please see the Supporting Information.

### One-pot reactions

One-pot reaction loading the unnatural substrates onto mACP was performed using CoaA, CoaD, CoaE, and Sfp. The reaction was performed in 50 mM Tris-HCl buffer pH 7.5, containing 200 mM NaCl, 0.5 mM DTT, and 5% glycerol, with 0.1 mM *apo*-mACP, 0.2 mM crosslinker, 12.5 mM MgCl_2_, 1 mM DTT, 8 mM ATP, 0.1 μM CoaA, 0.1 μM CoaD, 0.1 μM CoaE, and 0.2 μM Sfp, at 23 °C overnight. The resulting mixture was applied to a Superdex 75 16/600 Gl column (Cytiva) to remove the excess crosslinker and enzymatic proteins. The column was eluted with 50 mM Tris-HCl buffer pH 7.5, containing 200 mM NaCl, 0.5 mM DTT, and 5% glycerol, with a flow rate of 0.5 ml/min. Fractions were checked by 12% SDS-PAGE analyses, and pure fractions were collected and pooled.

### Acylation reactions

Malonyl-CoA and different fatty acids were prepared as stock solutions at 50 mM in DMSO. Acylation reaction loading the malonyl onto mACP was performed using Sfp. The reaction was performed in 50 mM Tris-HCl buffer pH 7.5, containing 200 mM NaCl, 0.5 mM DTT, and 5% glycerol, with 0.1 mM *apo*-mACP, 12.5 mM MgCl_2_, and 0.2 μM Sfp, at 23 °C overnight. The resulting mixture was applied to a Superdex 75 16/600 Gl column (Cytiva) to remove the excess malonyl-CoA and Sfp. The column was eluted with 50 mM Tris-HCl buffer pH 7.5, containing 200 mM NaCl, 0.5 mM DTT, and 5% glycerol, with a flow rate of 0.5 ml/min. Fractions were checked by 12% SDS-PAGE analyses, and pure fractions were collected and pooled; Acylation reaction loading the fatty acid onto mACP was performed using AasS. The reaction was performed in 50 mM Tris-HCl buffer pH 7.5, containing 200 mM NaCl, 0.5 mM DTT, and 5% glycerol, with 0.1 mM *holo*-mACP, 12.5 mM MgCl_2_, 0.5 mM TCEP, 10 mM ATP, and 2 μM AasS, at 23 °C overnight. The resulting mixture was applied to a Superdex 75 16/600 Gl column (Cytiva) to remove the excess octanoic acid and AasS. The column was eluted with 50 mM Tris • HCl buffer pH 7.5, containing 200 mM NaCl, 0.5 mM DTT, and 5% glycerol, with a flow rate of 0.5 ml/min. Fractions were checked by 12% SDS-PAGE analyses, and pure fractions were collected and pooled.

### Isothermal titration calorimetry (ITC)

ITC measurements were performed on a TA Instruments Affinity ITC LV at 25 °C using buffer-matched samples. Proteins were prepared in 50 mM Tris-HCl buffer pH 7.5, containing 150 mM NaCl. Acyl-mACP (0.01 mM; 350 μl) was loaded into the sample cell and titrated with LYRM2 (0.10 mM) in the syringe using 20 injections of 5 μl each at 0.5 μl/s injection rate, with a spacing time of 220 s and a stirring speed of 150 rpm. A matched LYRM2-into-buffer titration was collected under identical conditions and used as a blank to correct for heats of dilution and mixing. Integrated heats were analyzed in NanoAnalyze. Because the experiments were performed in a low-c regime and unconstrained fitting produced coupling between stoichiometry (n) and affinity, fits were performed with n fixed at 1.0 to improve convergence and enable comparison across the acyl series; dissociation constants are therefore reported as apparent values. All titrations were performed in duplicate, and values are reported as the mean and standard deviation unless otherwise noted.

## Data availability

All data generated or analyzed during this study are included in this published article and its supporting information files. Raw data is available upon request.

## Supporting information

This article contains supporting information (27, 28, 29, 30, 31, 32).

## Conflict of interest

The authors declare that they have no conflicts of interest with the contents of this article.
