# Supplementary material for: Selective recognition of mitochondrial acyl carrier protein by the regulatory protein LYRM2
Source: J Biol Chem. 2026 Aug 10;302(10):113422. doi: 10.1016/j.jbc.2026.113422 (PMC13579948; doi:10.1016/j.jbc.2026.113422)
Supplement: Supplementary file 1 [file mmc1.pdf]

Supporting Information

Selective recognition of mitochondrial acyl carrier protein by the regulatory protein LYRM2

Yixing Suo, James J. La Clair, and Michael D. Burkart*

Department of Chemistry and Biochemistry, University of California, San Diego,

9500 Gilman Drive, La Jolla, CA 92093-0358, United States

*To whom correspondence should be addressed: Michael D. Burkart (mburkart@ucsd.edu)

Table of Contents

A. General materials and methods.	S3
B. Protein expression and purification.	S3-S4
C. Boltz-2 covalent docking	S5-S6
D. Additional references.	S7
Supporting Figure S1	S8
Supporting Figure S2	S9
Supporting Figure S3	S10
Supporting Figure S4	S11
Supporting Figure S5	S12
Supporting Figure S6	S13
Supporting Figure S7	S14
Supporting Figure S8	S15
Supporting Figure S9	S16
Supporting Figure S10	S17
Supporting Table S1	S18
Supporting Table S2	S19
Supporting Table S3	S20
Supporting Table S4	S21
Supporting Table S5	S22

A. General materials and methods. All chemicals and reagents were purchased from commercial sources at 98% purity or greater. Competent *E. coli* cells, Ni-NTA resin, molecular weight markers, and SDS were obtained from Thermo Fisher Scientific. Acrylamide, Tris • HCl, isopropyl β-D-1-thiogalactopyranoside (IPTG), glycerol, dithiothreitol (DTT), ampicillin, and inorganic salts were obtained from Fisher Scientific. Malonyl coenzyme A lithium salt and imidazole were obtained from Sigma-Aldrich. Luria Broth (LB) and Agar were obtained from Research Product International. Kanamycin was obtained from Indofine. Milli-Q water was used for all experiments. All mixtures of liquids are provided in v:v unless otherwise noted.

B. Protein expression and purification.

The human mitochondrial regulatory protein LYRM2. LYRM2 gene fragment (aa 20-88, accession ID: Q9NU23) was synthesized by Twist Bioscience. The fragment was cloned into pET-28 by double site (NdeI + XhoI) restriction digestion to generate the expression vector with a previously engineered *N*-terminal Tev cleavable His_6-tag. The vector was transformed into *E. coli* BL21 (DE3) cells. Cells were grown in LB media with 50 mg/L kanamycin before induction with 0.5 mM IPTG at OD_{600} of 0.8 and incubated with shaking at 16 °C for 16-18 h. Cells were pelleted in a JLA-8.1 rotor at 2000 rpm, and resuspended and lysed by sonication in lysis buffer (50 mM Tris • HCl buffer pH 7.5, containing 150 mM NaCl). The resulting solution was centrifuged to give a clear supernatant, which was loaded onto column containing Ni-NTA resin (0.3 mL/L of culture). The column was washed with 5 column volumes (CV) of lysis buffer followed by lysis buffer containing 20 mM imidazole. Lysis buffer containing 200 mM imidazole was used to elute LYRM2. Dialysis using 3 kDa dialysis tubing (SnakeSkin) was performed in lysis buffer (2 L) at 4 °C overnight before being concentrated, flash frozen and stored at -80 °C.

The human mitochondrial acyl carrier protein (mACP). mACP was expressed and purified as previously published.[S1] Briefly, mACP with an *N*-terminal His_6-tag was chemically transformed into *E. coli* BL21 (DE3) cells. Cells were grown in LB media with 50 mg/L kanamycin before induction with 0.5 mM IPTG at OD_{600} of 0.8 and incubated with shaking at 16 °C for 16-18 h. Cells were pelleted in a JLA-8.1 rotor at 2000 rpm, and resuspended and lysed by sonication in lysis buffer (50 mM Tris • HCl buffer pH 7.5, containing 200 mM NaCl, 0.5 mM DTT, and 5% glycerol). The resulting solution was centrifuged to give a clear supernatant, which was loaded onto column containing Ni-NTA resin (0.3 mL/L of culture). The column was washed with 5 column volumes (CV) of lysis buffer followed by lysis buffer containing 20 mM imidazole. Lysis

buffer containing 200 mM imidazole was used to elute mACP. Dialysis using 3 kDa dialysis tubing (SnakeSkin) was performed in lysis buffer (2 L) at 4 °C overnight before being concentrated, flash frozen and stored at -80 °C.

The *Pseudomonas aeruginosa* ACP hydrolase (AcpH). AcpH was expressed and purified as previously published.[S2] Briefly, AcpH with an *N*-terminal His_6-tag was chemically transformed into *E. coli* BL21 (DE3) cells. Cells were grown in LB media with 50 mg/L kanamycin before induction with 0.5 mM IPTG at OD_{600} of 0.8 and incubated at 16 °C for 16-18 h. Cells were pelleted in a JLA-8.1 rotor at 2000 rpm, and re-suspended and lysed by sonication in lysis buffer (50 mM Tris • HCl buffer pH 7.5, containing 500 mM NaCl, and 10 % glycerol). The resulting solution was centrifuged to give a clear supernatant, which was loaded onto column containing Ni-NTA resin (0.3 mL/L of culture). The column was washed with 5 column volumes (CV) of lysis buffer followed by lysis buffer containing 20 mM imidazole. Lysis buffer containing 200 mM imidazole was used to elute AcpH. Dialysis using 10 kDa dialysis tubing (SnakeSkin) was performed in lysis buffer (2 L) at 4 °C for 2 h before being flash frozen and stored at -80 °C.

The coenzyme A biosynthetic proteins CoaA, CoaD, and CoaE. CoaA, CoaD, CoaE were expressed and purified as previously published.[S3] Briefly, CoaA, CoaD, and CoaE with *N*-terminal MBP-tags were chemically transformed into *E. coli* BL21 (DE3) cells, separately. Cells were grown in LB media with 100 mg/L ampicillin before induction with 1 mM IPTG at OD_{600} of 0.8 and incubated with shaking at 16 °C for 16-18 h. Cells were pelleted in a JLA-8.1 rotor at 2000 rpm, and re-suspended and lysed by sonication in lysis buffer (50 mM potassium phosphate pH 7.5 containing 150 mM NaCl). The resulting solution was centrifuged to give a clear supernatant, which was loaded onto column containing amylose resin. The column was washed with 5 column volumes (CV) of the lysis buffer. Lysis buffer containing 10 mM of maltose was used to elute CoaA, CoaD, and CoaE, separately. Dialysis using 10 kDa dialysis tubing (SnakeSkin) was performed in lysis buffer (2 L) at 4 °C overnight before being concentrated, flash frozen and stored at -80 °C.

The *Bacillus subtilis* phosphopantetheinyl transferase (Sfp). Sfp was expressed and purified as previously published.[S3] Briefly, Sfp with an *N*-terminal His_6-tag was chemically transformed into *E. coli* BL21 (DE3) cells. Cells were grown in LB media with 50 mg/L kanamycin before induction with 1 mM IPTG at OD_{600} of 0.8 and incubated with shaking at 16 °C for 16-18 h. Cells were

pelleted in a JLA-8.1 rotor at 2000 rpm, and re-suspended and lysed by sonication in lysis buffer (50 mM Tris • HCl buffer pH 7.5, containing 150 mM NaCl). The resulting solution was centrifuged to give a clear supernatant, which was loaded onto column containing Ni-NTA resin (0.3 mL/L of culture). The column was washed with 5 column volumes (CV) of the lysis buffer followed by lysis buffer containing 20 mM imidazole. Lysis buffer containing 200 mM imidazole was used to elute Sfp. Dialysis using 10 kDa dialysis tubing (SnakeSkin) was performed in lysis buffer (2 L) at 4 °C overnight before being concentrated, flash frozen and stored at -80 °C.

The *Vibrio harveyi* acyl acyl-carrier protein synthetase (AasS). AasS was expressed and purified as previously published.[S4] Briefly, AasS with an *N*-terminal His_6-tag was chemically transformed into *E. coli* BL21 (DE3) cells. Cells were grown in LB media with 50 mg/L kanamycin before induction with 0.5 mM IPTG at OD_{600} of 0.8 and incubated with shaking at 16 °C for 16-18 h. Cells were pelleted in a JLA-8.1 rotor at 2000 rpm, and re-suspended and lysed by sonication in lysis buffer (50 mM Tris • HCl buffer pH 7.5, containing 150 mM NaCl). The resulting solution was centrifuged to give a clear supernatant, which was loaded onto column containing Ni-NTA resin (0.3 mL/L of culture). The column was washed with 5 column volumes (CV) of the lysis buffer followed by lysis buffer containing 20 mM imidazole. Lysis buffer containing 200 mM imidazole was used to elute AasS. Dialysis using 10 kDa dialysis tubing (SnakeSkin) was performed in lysis buffer (2 L) at 4 °C overnight before being concentrated, flash frozen and stored at -80 °C.

C. Boltz-2 covalent docking of acyl-phosphopantetheine variants on mACP against LYRM2. Structure and binding-affinity predictions were generated with Boltz-2 (v2.2.1).[S5] Boltz-2 uses AlphaFold3 deep-learning design and adds physics guides to accurately dock biomolecules. Each modeled system comprised three components: mACP (Uniprot NDUFAB1; chain A; 99 residues derived from PDB: 2DNW), LYRM2 (UniProt Q9NU23; chain B; 88 residues), and an acyl-phosphopantetheine (acyl-PPant) ligand (chain L). The PPant arm was tethered covalently to the mACP by an explicit bond constraint between the active serine side-chain oxygen (OG) of chain A and the ligand phosphorus atom. Five ligand variants were modeled, differing in acyl chain length across the even-carbon series C8, C10, C12, C14 and C16 (octanoyl, decanoyl, dodecanoyl, myristoyl and hexadecanoyl). Each variant was supplied to Boltz-2 as a ligand SMILES string. Multiple-sequence alignments were generated using the Boltz MSA server (ColabFold).[S6] For each variant, five diffusion samples were generated with three recycling steps using the reference

PyTorch attention implementation and the expandable-segments CUDA allocator. The top-ranked sample by Boltz confidence was taken as the representative pose for each variant; all reported metrics were confirmed to be stable across the five samples. For each model, we recorded the interface predicted TM-score for the LYRM2·mACP (ipTM), the ligand and protein interface pTM values, the mean predicted Local Distance Difference Test of the complex (pLDDT), and the Boltz-2 affinity-module outputs (more negative values indicate predicted stronger binding interpreted as a relative ranking within the covalent acyl series). Interface residues were defined as any residue with at least one atom within 5 Å of the partner chain; the Ser49:OG–P bond length was measured in each output model as a geometric validity check.

D. Additional references

S1. Suo, Y.; Chen, A.; La Clair, J. J.; Burkart, M. D. (2023) Substrate Sequestration and Chain Flipping in Human Mitochondrial Acyl Carrier Protein. *Biochem.* **62**, 3548-3553.

S2. Kosa, N. M.; Pham, K. M.; Burkart, M. D. (2014) Chemoenzymatic Exchange of Phosphopantetheine on Protein and Peptide. *Chem. Sci.* **5**, 1179-1186.

S3. Worthington, A. S.; Burkart, M. D. (2006) One-Pot Chemo-Enzymatic Synthesis of Reporter-Modified Proteins. *Org. Biomol. Chem.* **4**, 44-46.

S4. Sztain, T.; Bartholow, T. G.; McCammon, J. A.; Burkart, M. D. (2019) Shifting the Hydrolysis Equilibrium of Substrate Loaded Acyl Carrier Proteins. *Biochem.* **58**, 3557-3560.

S5. Passaro, S.; Corso, G.; Wohlwend, J.; Reveiz, M.; Thaler, S.; Somnath, V. R.; Getz, N.; Portnoi, T.; Roy, J.; Stark, H.; Kwabi-Addo, D.; Beaini, D.; Jaakkola, T.; Barzilay, R. (2025) Boltz-2: Towards Accurate and Efficient Binding Affinity Prediction. bioRxiv p 2025.06.14.659707.

S6. Mirdita, M.; Schütze, K.; Moriwaki, Y.; Heo, L.; Ovchinnikov, S.; Steinegger, M. (2022) ColabFold: Making Protein Folding Accessible to All. *Nat. Methods* **19**, 679-682.

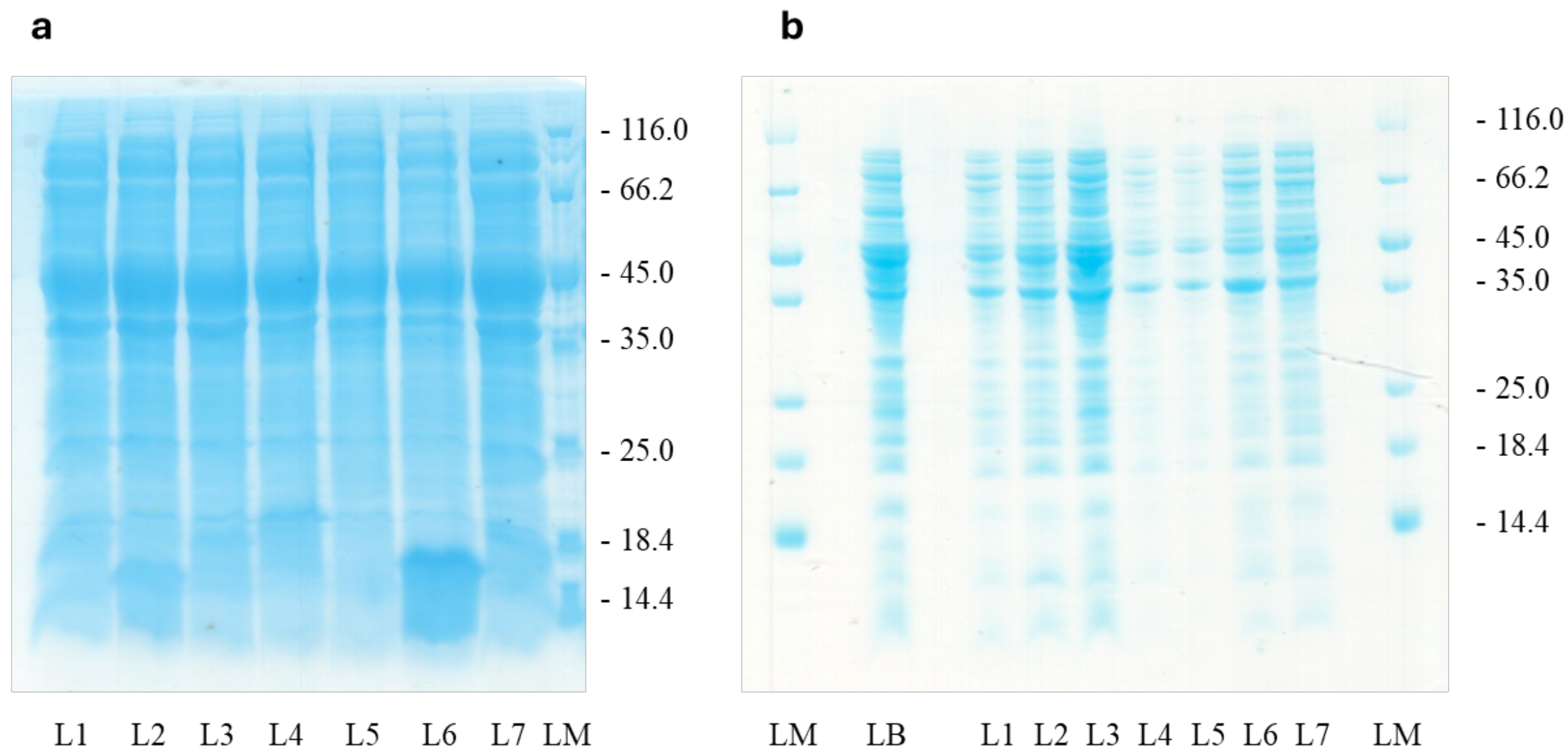

Supporting Figure S1. LYRM proteins independent expression trail. The cells were lysed by BugBuster Protein Extraction Reagent. **(a)** Whole cell lysate shown by 12% SDS-PAGE analysis. Lanes are as follows: (L1) LYRM1, (L2) LYRM2, (L3) LYRM5, (L4) LYRM7, (L5) LYRM8, (L6) ACN9, (L7) FMC1, (LM) lane marker. **(b)** Soluble protein in the supernatant after centrifugation shown by 12% SDS-PAGE analysis. Lanes are as follows: (LM) lane marker, (LB) blank *E. coli* control, (L1) LYRM1, (L2) LYRM2, (L3) LYRM5, (L4) LYRM7, (L5) LYRM8, (L6) ACN9, (L7) FMC1, (LM) lane marker.

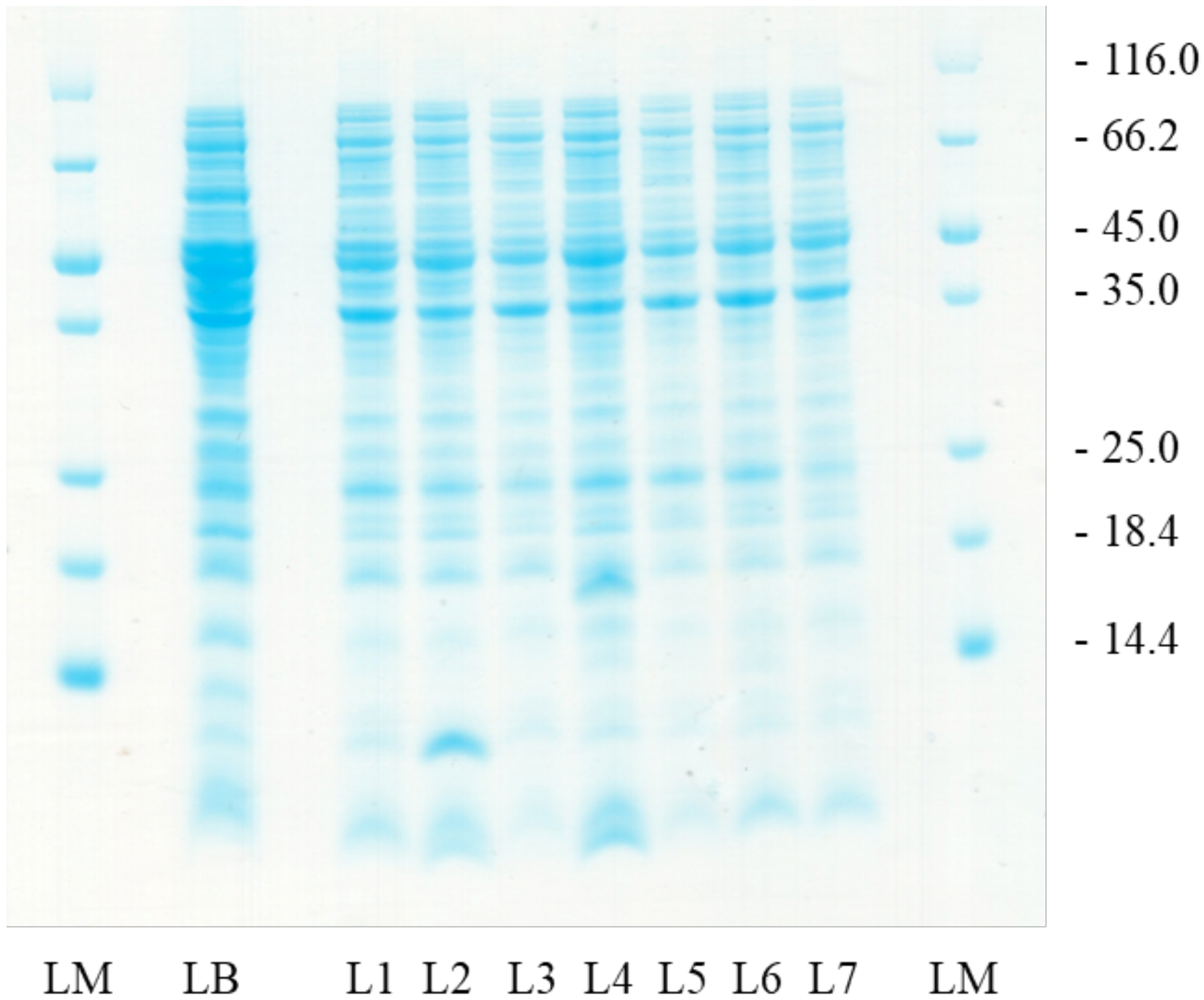

Supporting Figure S2. LYRM proteins co-expression trail with mACP. The cells were lysed by BugBuster® Protein Extraction Reagent. Soluble protein in the supernatant after centrifugation shown by 12% SDS-PAGE analysis. Lanes are as follows: (LM) lane marker, (LB) blank *E. coli* control, (L1) LYRM1, (L2) LYRM2, (L3) LYRM5, (L4) LYRM7, (L5) LYRM8, (L6) ACN9, (L7) FMC1, (LM) lane marker.

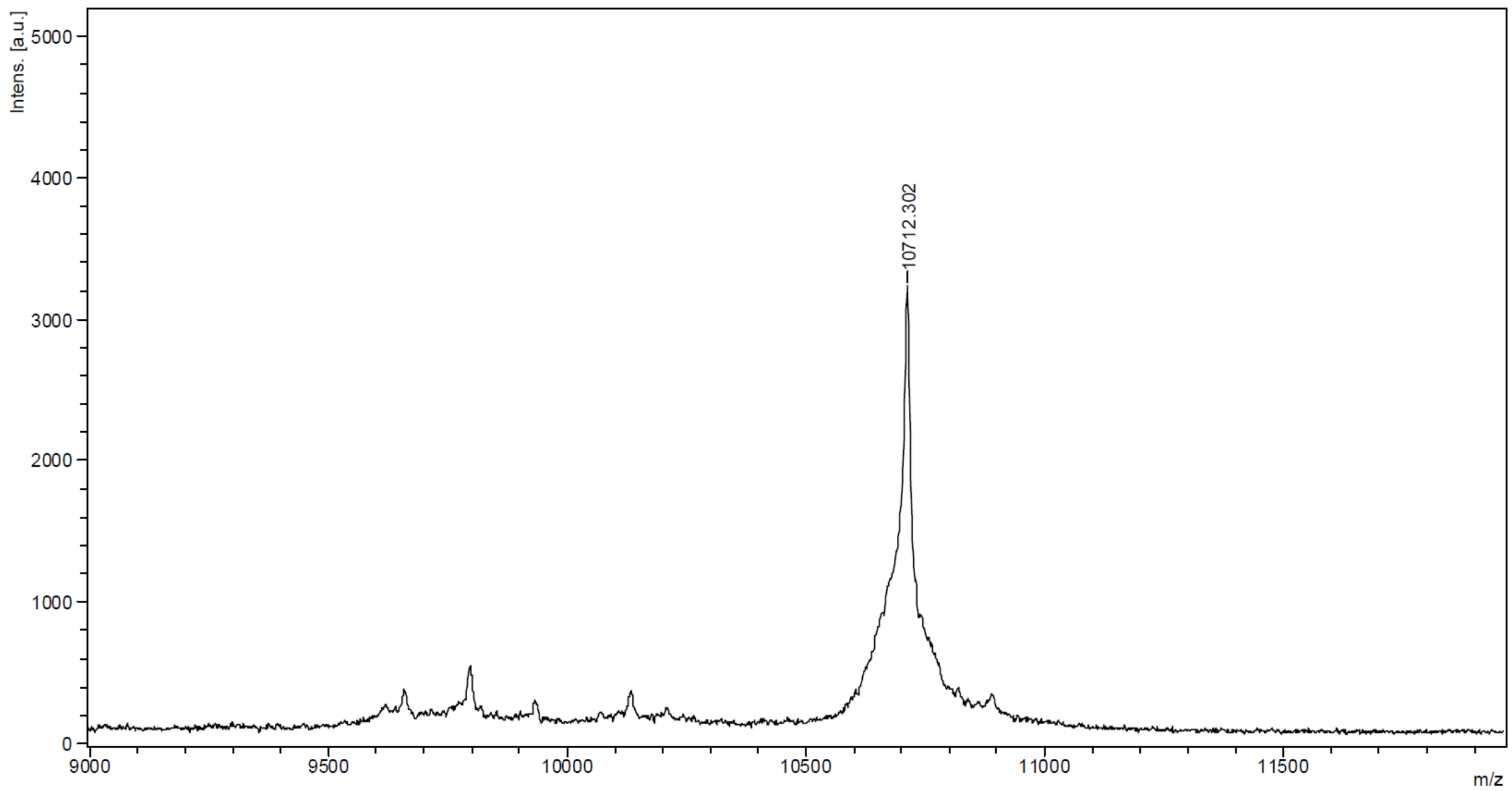

Supporting Figure S3. MALDI-TOF mass spectrometry of His_6-LYRM2. The mass spectrum indicates the N-terminal methionine excision in *E. coli*.

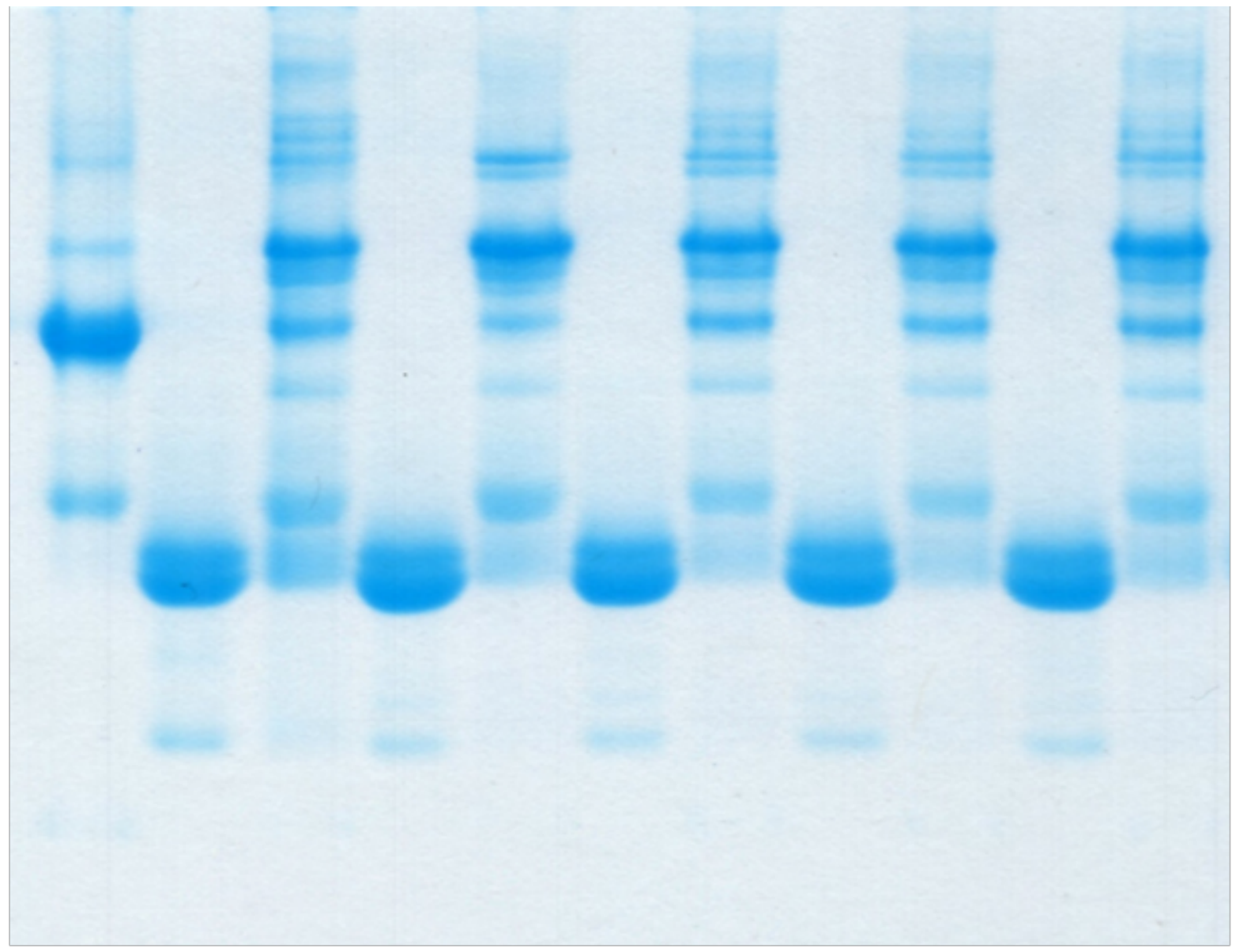

LYRM2	+	-	+	-	+	-	+	-	+	-	+
C8-mACP	-	+	+	-	-	-	-	-	-	-	-
C10-mACP	-	-	-	+	+	-	-	-	-	-	-
C12-mACP	-	-	-	-	-	+	+	-	-	-	-
C14-mACP	-	-	-	-	-	-	-	+	+	-	-
C16-mACP	-	-	-	-	-	-	-	-	-	+	+

Supporting Figure S4. 15% Urea-PAGE of LYRM2 interacts with different acyl-mACPs. New bands show up when LYRM2 incubating with acyl-mACP, indicating the new complex formation and stability even under mild denature conditions.

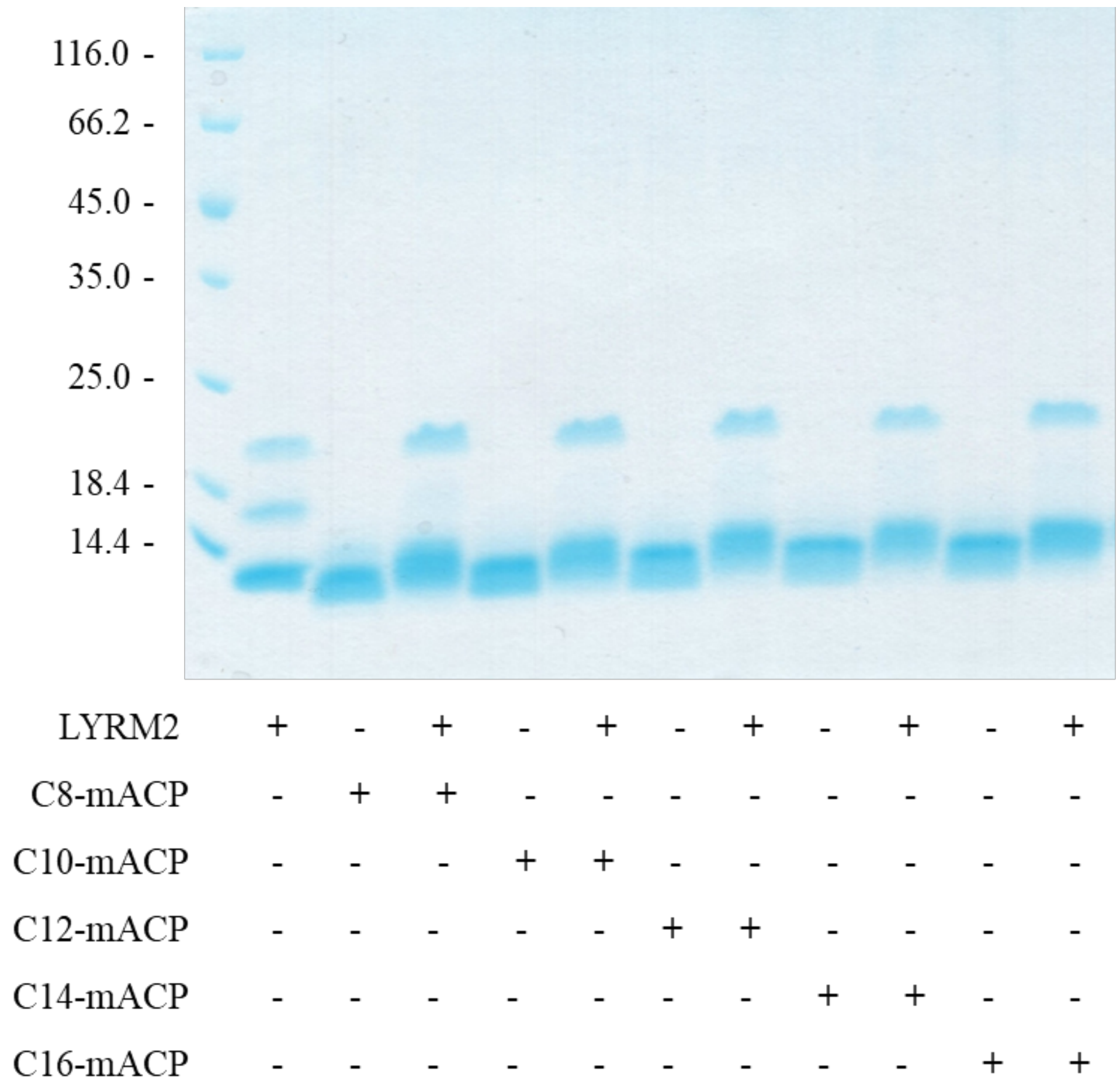

Supporting Figure S5. 12% SDS-PAGE of LYRM2 interacts with different acyl-mACPs.

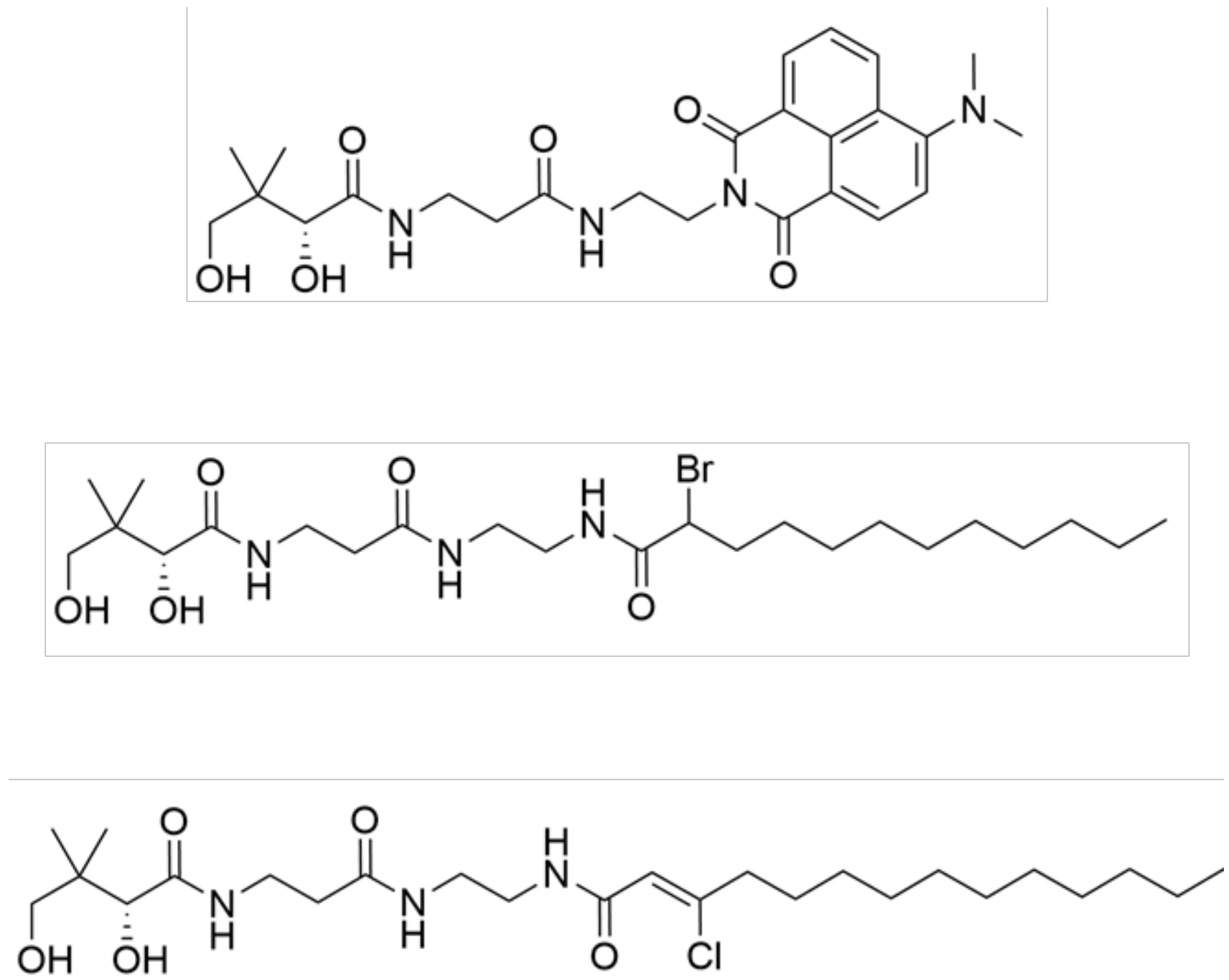

Supporting Figure S6. Non-native substrate structures. From top to bottom: 4-*N,N*-dimethylamino-1,8-naphthalimiden pantetheinamide (4-DMN), C12-α-bromoamide pantetheinamide (C12αBr), C14-*Z*-chloroacrylate pantetheinamide (C14βCl). The probes were loaded on *apo*-mACP through 'one-pot' reaction as described above.

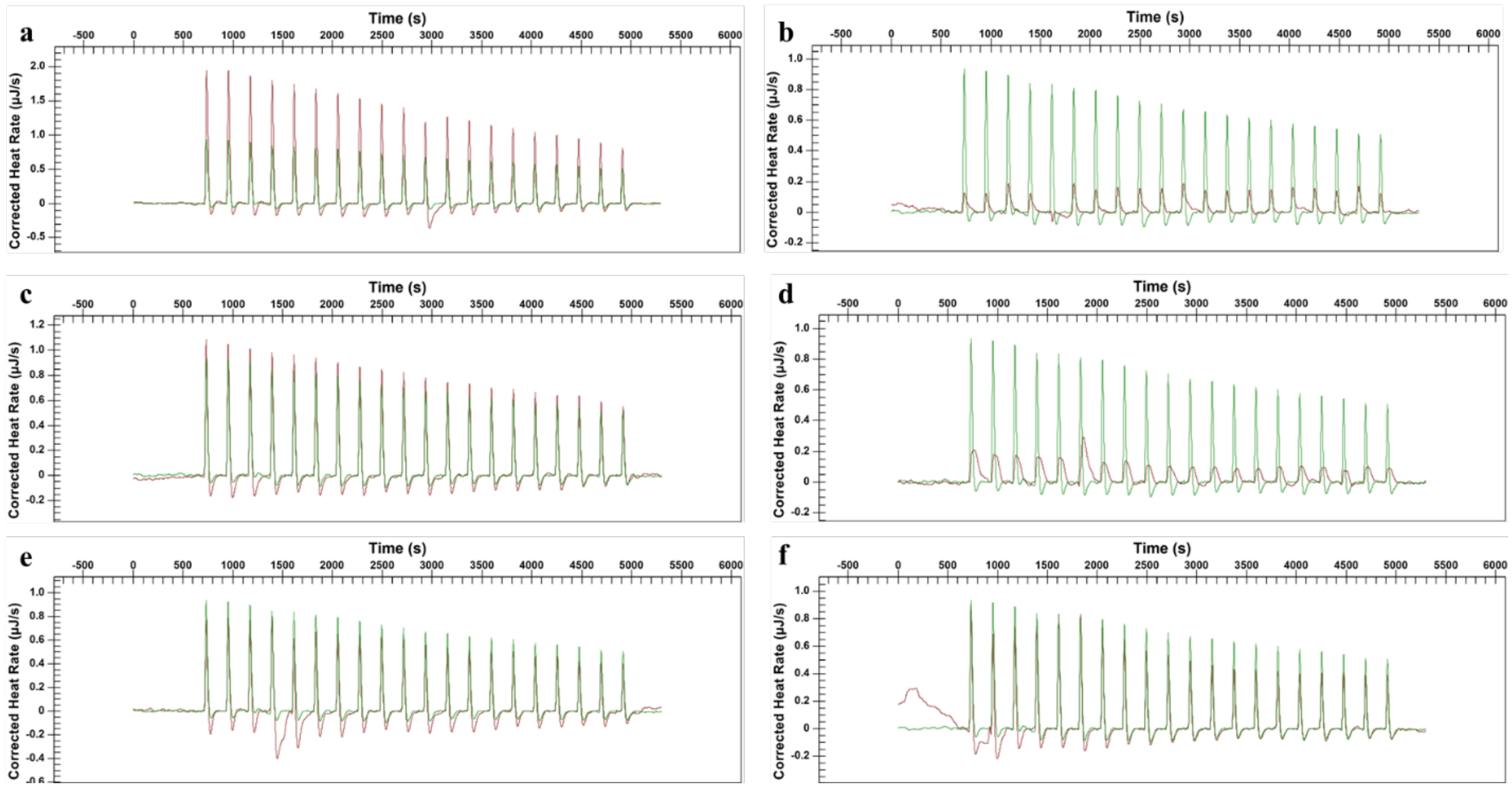

Supporting Figure S7. Second representative ITC injection traces for LYRM2 titrated into the acyl-mACP series. **(a)** C8-mACP **(b)** C10-mACP **(c)** C12-mACP **(d)** C14-mACP **(e)** C16-mACP **(f)** malonyl-mACP. LYRM2-into-acyl-mACP runs (red) are overlaid against LYRM2-into-buffer blank (green).

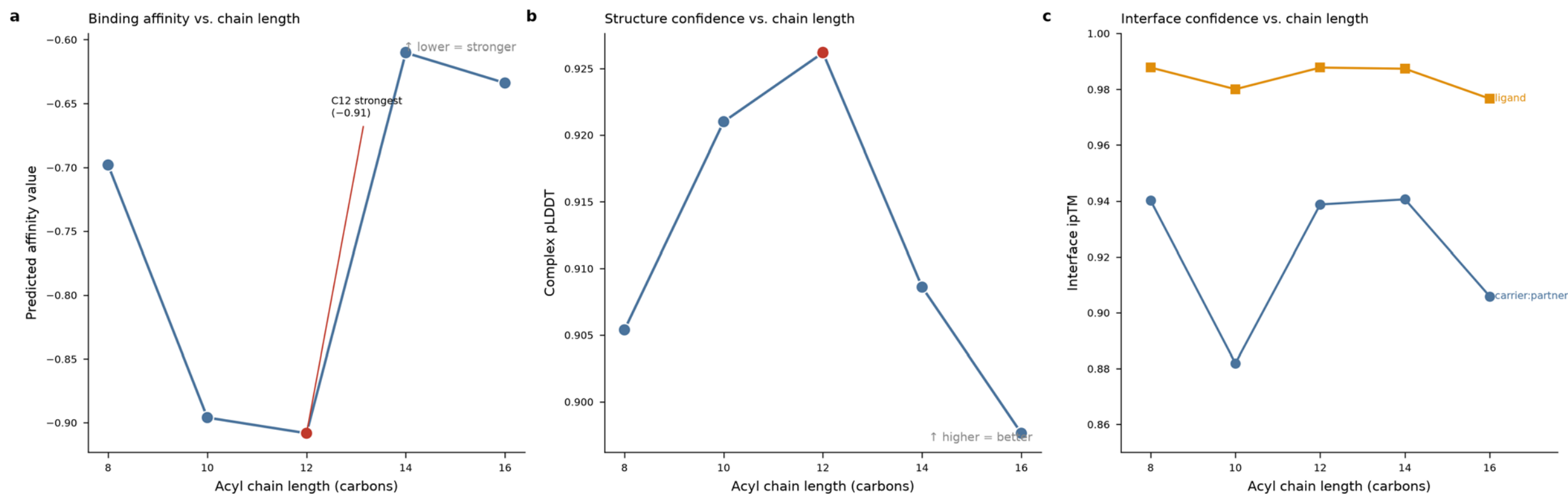

Supporting Figure S8. Boltz-2 prediction of LYRM2 binding across the acyl-mACP series. Predicted binding affinity (more negative values indicate stronger predicted binding), complex confidence (pLDDT), interface confidence (ipTM), and ligand interface confidence are shown for the top-ranked model of each acyl-mACP complex (C8-C16). All complexes were predicted with consistently high confidence, while the predicted affinity displayed a non-monotonic dependence on acyl chain length, with the strongest predicted interaction observed for C12-mACP and a broader optimum spanning C10-C12. Numerical prediction metrics are summarized in Supporting Tables S4 and S5.

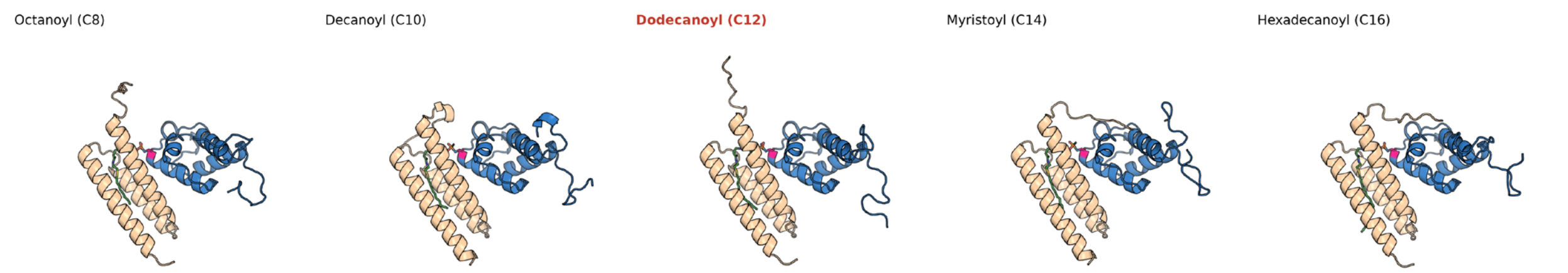

Supporting Figure S9. Representative models of LYRM2 bound to acyl-mACP carrying C8-C16 acyl chains. Complexes are shown in a common orientation after superposition of the mACP backbone. LYRM2 is shown in wheat, mACP in blue, the acyl-phosphopantetheine ligand in green sticks, and the covalent attachment residue Ser49 in magenta. The imposed Ser49-phosphopantetheine linkage was maintained in all predicted models, demonstrating successful covalent docking across the acyl series. The acyl tail extends progressively deeper into the LYRM2 helical bundle with increasing chain length.

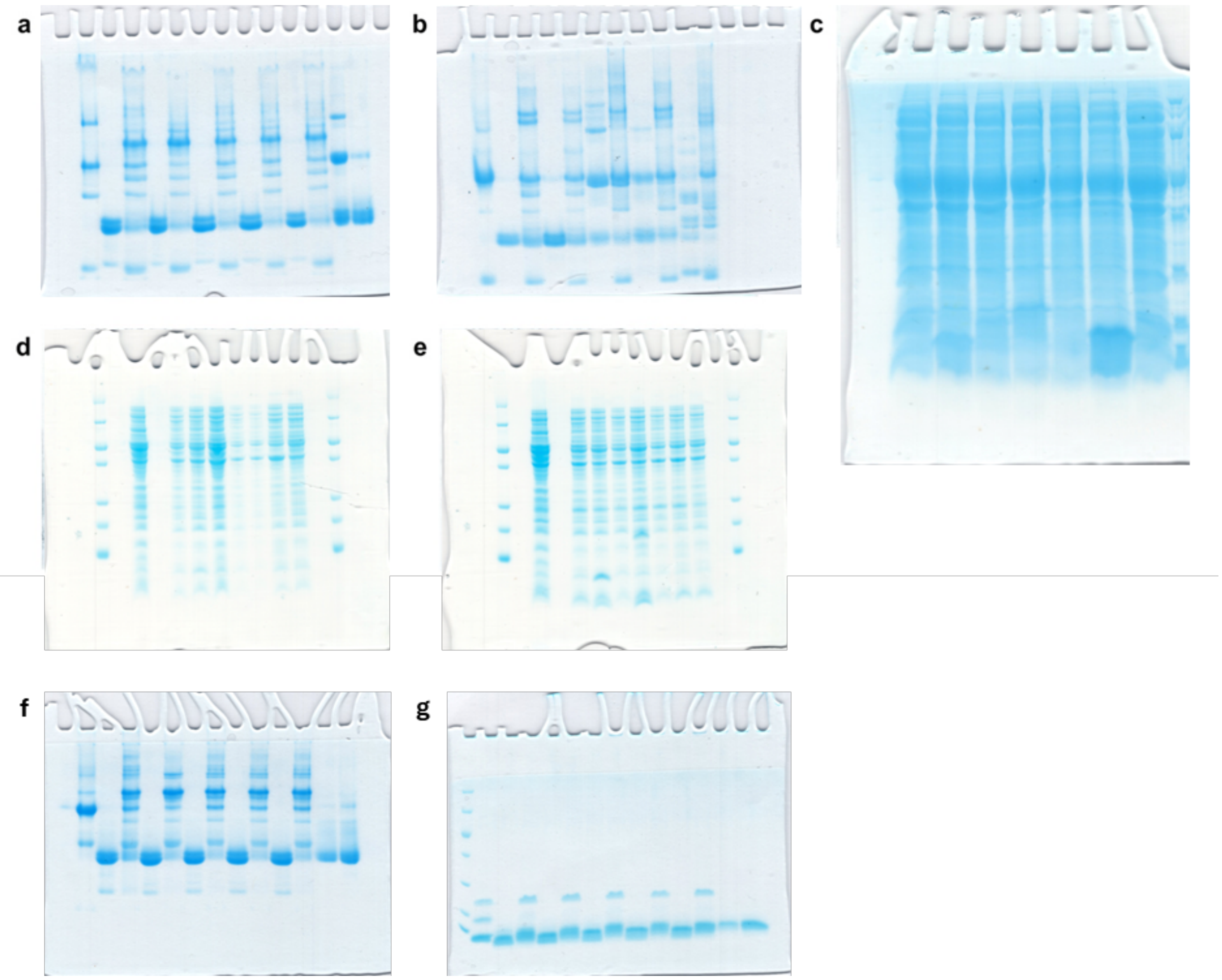

Supporting Figure S10. Uncropped gels used in the manuscript. **(a)** Fig. 2a. **(b)** Fig. 2b. **(c)** Supporting Fig. S1a. **(d)** Supporting Fig. S1b. **(e)** Supporting Fig. S2. **(f)** Supporting Fig. S4. **(g)** Supporting Fig. S5.

Supporting Table S1. LYRM protein functions and structures.

	Function	Structure	PDB
**LYRM1**	Implicated in insulin signaling	No reported structure	
**LYRM2**	Promotes integration of complex I	No reported structure	
**LYRM3**	Structural subunit of complex I	Present in complex I cryo-EM	5XTD
**LYRM4**	Involved in Fe-S cluster biogenesis	Crystal structure of mACP-ISD11	6ODD
**LYRM5**	Regulates electron transfer flavoprotein	No reported structure	
**LYRM6**	Structural subunit of complex I	Present in complex I cryo-EM	5XTD
**LYRM7**	Assembly factor of complex III	No reported structure	
**LYRM8**	Assembly factor of complex II	No reported structure	
**LYRM9**	Unknown	No reported structure	
**ACN9**	Assembly factor of complex II	No reported structure	
**FMC1**	Assembly factor of complex V	No reported structure	
**L0R8F8**	Involved in mitoribosome/complex I biogenesis	Present in mt-LSU assembly intermediate cryo-EM	7ODR

Supporting Table S2. LYRM protein cloning sequences.

	Amino acid sequence
**LYRM1**	MGSSHHHHHHSSGENLYFQGHMTTATRQEVLGLYRSIFRLARKWQATSGQMEDTIKEKQYILNEARTLFRKNKNLTDTDLIKQCIDECTARIEIG LHYKIPYPRPIHLPPMGLTPLRGRGLRSQEKLRKLSKPVYLRSHDEVS*
**LYRM2**	MGSSHHHHHHSSGENLYFQGHMQQVLLLYRRILQTIRQVPNDSDRKYLKDWAREEFRRNKSATEEDTIRMMITQGNMQLKELEKTLALAKS*
**LYRM5**	MGSSHHHHHHSSGENLYFQGHMKMANSLRGEVLKLYKNLLYLGRDYPKGADYFKKRLKNIFLKNKDVKNPEKIKELIAQGEFVMKELEALYFLRK YRAMKQRYYSDTNKTN*
**LYRM7**	MGSSHHHHHHSSGENLYFQGHMGRAVKVLQLFKTLHRTRQQVFKNDARALEAARIKINEEFKNNKSETSSKKIEELMKIGSDVELLLRTSVIQGI HTDHNTLKLVPRKDLLVENVPYCDAPTQKQ*
**LYRM8**	MGSSHHHHHHSSGENLYFQGHMSRHSRLQRQVLSLYRDLLRAGRGKPGAEARVRAEFRQHAGLPRSDVLRIEYLYRRGRRQLQLLRSGHATA MGAFVRPRAPTGEPGGVGCQPDDGDSPRNPHDSTGAPETRPDGR*
**ACN9**	MGSSHHHHHHSSGENLYFQGHMGDQYVKDEFRRHKTVGSDEAQRFLQEWEVYATALLQQANENRQNSTGKACFGTFLPEEKLNDFRDEQI GQLQELMQEATKPNRQFSISESMKPKF*
**FMC1**	MGSSHHHHHHSSGENLYFQGHMAALGSPSHTFRGLLRELRYLSAATGRPYRDTAAYRYLVKAFRAHRVTSEKLCRAQHELHFQAATYLCLLRSIR KHVALHQEFHGKGERSVEESAGLVGLKLPHQPGGKGWEP*

Supporting Table S3. ITC titrations binding parameters (n fixed at 1).

	K_d		Avg	Std	c
**malonyl**	2.83E-05	2.66E-05	2.74E-05	8.70E-07	0.4
**C8**	9.83E-06	9.64E-06	9.74E-06	9.15E-08	1.0
**C10**	2.25E-05	1.63E-06	1.21E-05	1.04E-05	0.8
**C12**	2.64E-06	2.37E-06	2.50E-06	1.38E-07	4.0
**C14**	8.49E-07	8.59E-07	8.54E-07	4.95E-09	11.7
**C16**	5.19E-07	1.13E-05	5.91E-06	5.40E-06	1.7

Supporting Table S4. Acyl-phosphopantetheine ligands used for Boltz-2 covalent docking. Five acyl-PPant ligands (C8-C16) differing only in acyl chain length were covalently attached to mACP for structure prediction. Heavy-atom counts were verified from the SMILES with RDKit.

Variant	Acyl	Ligand heavy atoms	Attachment atom	SMILES
**Octanoyl**	C8	31	P68	O=C(SCCNC(CCNC([C@H](O)C(C)(C)COP([O-])([O-])=O)=O)=O)CCCCCCC
**Decanoyl**	C10	33	P74	O=C(SCCNC(CCNC([C@H](O)C(C)(C)COP([O-])([O-])=O)=O)=O)CCCCCCCCC
**Dodecanoyl**	C12	35	P80	O=C(SCCNC(CCNC([C@H](O)C(C)(C)COP([O-])([O-])=O)=O)=O)CCCCCCCCCCC
**Myristoyl**	C14	37	P86	O=C(SCCNC(CCNC([C@H](O)C(C)(C)COP([O-])([O-])=O)=O)=O)CCCCCCCCCCCCC
**Hexadecanoyl**	C16	39	P92	O=C(SCCNC(CCNC([C@H](O)C(C)(C)COP([O-])([O-])=O)=O)=O)CCCCCCCCCCCCCCC

Supporting Table S5. Boltz-2 prediction metrics for LYRM2·acyl-mACP complexes. Prediction statistics for the top-ranked model of each acyl-mACP complex, including predicted binding affinity, interface confidence (ipTM), ligand interface confidence, complex confidence (pLDDT), binding probability, and interface geometry. Values are representative of the top-ranked model, with ranges across five diffusion samples reported where applicable.

Variant	Acyl	ipTM (A:B)	ipTM range (5)	ligand ipTM	protei n	pLDDT	pLDDT range	Affinity	Bind prob.	Ser49:OG –P (Å)	Interface A	Interface B
**Octanoyl**	C8	0.94	0.901–0.940	0.988	0.944	0.905	0.893–0.905	-0.698	0.971	1.93	19	15
**Decanoyl**	C10	0.882	0.867–0.915	0.98	0.904	0.921	0.896–0.921	-0.896	0.983	2.02	20	14
**Dodecanoyl**	C12	0.939	0.878–0.939	0.988	0.949	0.926	0.901–0.926	-0.908	0.959	1.82	20	14
**Myristoyl**	C14	0.941	0.904–0.941	0.987	0.963	0.909	0.893–0.909	-0.61	0.976	2.2	29	19
**Hexadecanoyl**	C16	0.906	0.887–0.938	0.977	0.933	0.898	0.887–0.898	-0.634	0.972	2	28	22
